# Factors Associated with Axillary Lymph Node Status in Clinically Node-Negative Breast Cancer Patients Undergoing Neoadjuvant Chemotherapy

**DOI:** 10.3390/cancers14184451

**Published:** 2022-09-14

**Authors:** Chi-Chang Yu, Yun-Chung Cheung, Shir-Hwa Ueng, Yung-Chang Lin, Wen-Ling Kuo, Shih-Che Shen, Yung-Feng Lo, Shin-Cheh Chen

**Affiliations:** 1 Department of General Surgery, Chang Gung Memorial Hospital at Linkou, Chang Gung University, Taoyuan 333, Taiwan; 2 Department of Diagnostic Radiology, Chang Gung Memorial Hospital at Linkou, Chang Gung University, Taoyuan 333, Taiwan; 3 Department of Pathology, Chang Gung Memorial Hospital at Linkou, Chang Gung University, Taoyuan 333, Taiwan; 4 Department of Hematology-Oncology, Chang Gung Memorial Hospital at Linkou, Chang Gung University, Taoyuan 333, Taiwan

**Keywords:** axillary lymph node, breast cancer, neoadjuvant chemotherapy, node-negative, pathological response

## Abstract

**Simple Summary:**

Routine axillary surgery for lymph node staging is necessary for invasive breast cancer, according to current guidelines. However, advances in breast tumor biology and the in vivo tumor response to drugs provided by neoadjuvant chemotherapy (NAC) have led to the development of new effective drugs and higher rates of pathological complete response (pCR) in the breast or axilla. In this retrospective study, we aimed to investigate the factors influencing axillary lymph node (ALN) status after NAC in patients initially diagnosed with clinically node-negative (cN0) breast cancer. We found that pCR of the breast was a predictor of negative ALN status, and the presence of lymphovascular invasion was a predictor of positive ALN status. Our findings support the omission of axillary surgery in patients who achieve breast-pCR and provide the rationale for trials to investigate the feasibility of breast-conserving surgery without concurrent axillary surgery in patients who meet certain criteria.

**Abstract:**

Adequate axillary lymph node (ALN) staging is critical for patients with invasive breast cancer. However, neoadjuvant chemotherapy (NAC) was associated with a lower risk of ALN metastasis compared with those who underwent primary surgery among clinically node-negative (cN0) patients. This study aimed to investigate the factors associated with ALN status among patients with cN0 breast cancer undergoing NAC. A total of 222 consecutive patients with cN0 breast cancer undergoing NAC between January 2012 and December 2021 were reviewed. Univariate and multivariate analyses were performed to compare factors associated with positive ALN status. Seventeen patients (7.7%) had ALNs metastases. Here, 90 patients (40.5%) achieved pathologic complete response in the breast (breast-pCR), and all had negative ALN status. Lymphovascular invasion (odds ratio: 29.366, *p* < 0.0001) was an independent risk predictor of ALN metastasis in all study populations. Among patients without breast-pCR, mastectomies were performed more frequently in patients with ALN metastasis (52.9%) than in those without metastasis (20.9%) (*p* = 0.013). Our findings support the omission of axillary surgery in patients who achieve breast-pCR. Prospective studies are needed to confirm the feasibility of a future two-stage surgical plan for breast-conserving surgery in patients who are likely to achieve breast-pCR during clinical evaluation.

## 1. Introduction

Determining the status of the axilla in invasive breast cancer is critical because the metastasis of axillary lymph nodes (ALN) is closely related to the prognosis of breast cancer and significantly influences the treatment strategy [1,2,3]. Although axillary lymph node dissection (ALND) has traditionally been the standard procedure for axillary staging of breast cancer, it is still inevitably accompanied by a proportion of complications, including lymphedema, axillary paresthesia, pain, and shoulder movement restrictions, which have adverse effects on patients’ quality of life [4,5,6,7]. Based on the advantages of having a similar prognosis and less chance of complications compared with ALND, sentinel lymph node biopsy (SLNB), a minimally invasive procedure, has become an alternative to ALND for axillary staging in patients with clinically node-negative (cN0) breast cancer [8,9,10,11,12]. Although SLNB has significantly less frequent surgical complications than ALND, there is still a 2–6.9% risk of lymphedema, which not only affects upper-body function but also has an adverse influence on the quality of life of breast cancer patients [13,14,15]. Therefore, the development of a non-invasive examination to replace pathological examination for axillary staging is undoubtedly the most efficient way to avoid postoperative complications; however, such a well-recognized examination is still lacking.

For newly diagnosed breast cancers, adequate axillary staging prior to surgery or neoadjuvant therapy is required to select candidates for subsequent, less extensive axillary surgery. Several imaging modalities, including axillary ultrasound, magnetic resonance imaging (MRI), and ^18^F-fluorodeoxyglucose positron emission tomography/computed tomography (FDG-PET/CT), have been shown to have better sensitivity for axillary lymph node staging than physical examination [16,17,18,19]. Among these imaging modalities, axillary ultrasound has become a routine examination for axillary staging in many regions because of its ability to rapidly provide instant ultrasound-guided fine-needle aspiration cytology (FNAC). The sensitivity of axillary ultrasound combined with FNAC for axillary staging is approximately 80% [20,21].

The role of neoadjuvant chemotherapy (NAC) in breast cancer treatment has expanded from initially increasing the likelihood of surgery for inoperable tumors to increasing the likelihood of breast-conserving surgery (BCS) for operable tumors. In addition, providing patients with clinically node-positive (cN+) breast cancer and the opportunity to undergo de-escalation axillary surgery by SLNB. Moreover, NAC provides an opportunity to assess tumor response to drugs in vivo and escalate adjuvant therapy in those without achievement of a pathologic complete response (pCR) after NAC [22]. Based on the advantages mentioned above regarding NAC, clinical physicians are bound to face more patients with not only cN+ but also cN0 breast cancer receiving NAC.

In a general breast cancer population with cN0 disease, approximately 74% are sentinel lymph node (SLN) negative [23]. Several studies have demonstrated that in patients with cN0 breast cancer, the group receiving NAC had a significantly lower nodal positivity rate compared with the primary surgery group [24,25]. In addition, when cN0 breast cancer patients with specific biological characteristics such as triple-negative or human epidermal growth factor receptor 2 (HER2)-positive disease achieved breast pCR after receiving NAC, the postoperative lymph node-positive (ypN+) rate was even lower than 2% [26,27]. Therefore, regardless of tumor biology and response to drugs, the current practice of routine axillary surgery in cN0 breast cancer patients following NAC deserves reconsideration.

The primary objective of this study was to identify the specific clinicopathological factors that could serve as predictors of ALN status after NAC in patients with cN0 breast cancer. A secondary objective was to identify patients who might be treated with the omission of axillary surgery for ALN staging if the ypN+ rate was low.

## 2. Materials and Methods

### 2.1. Patients

**Figure 1:**
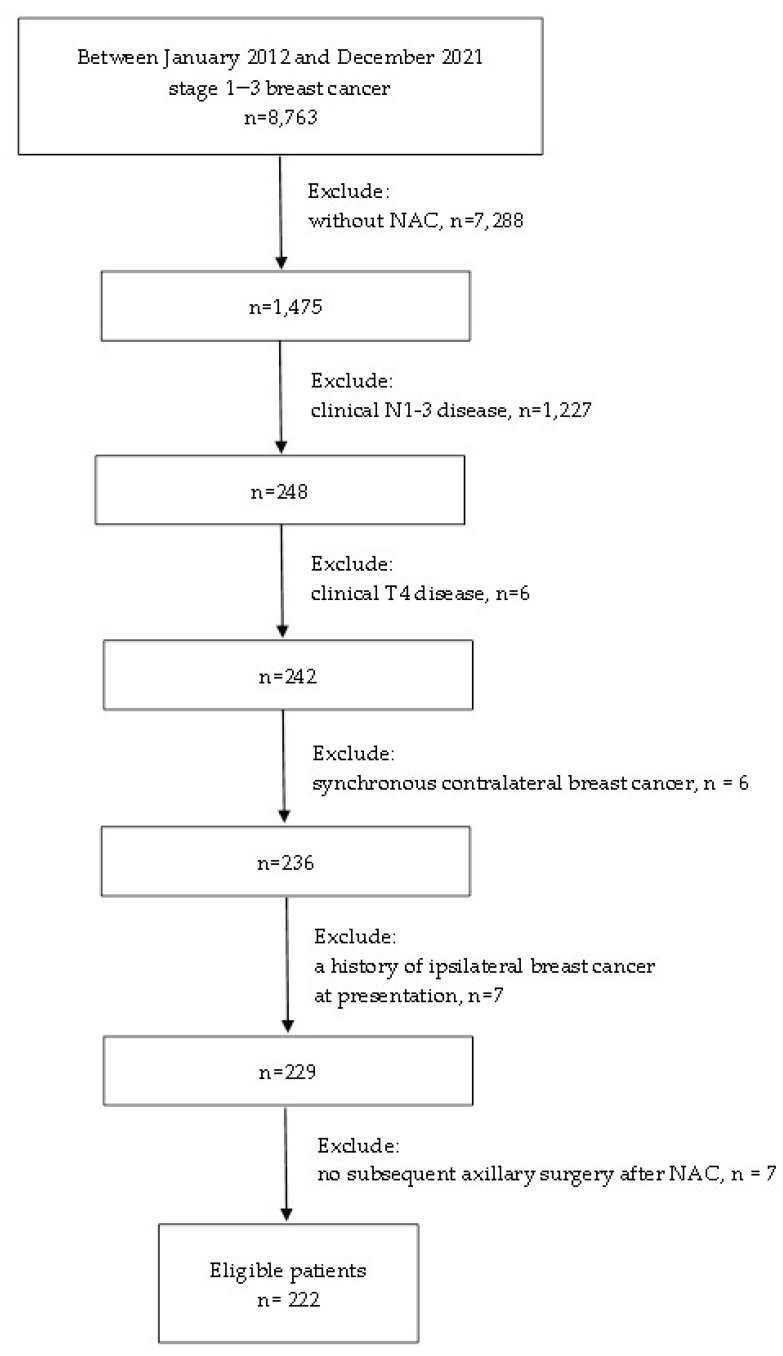
The flow diagram of the study. NAC, neoadjuvant chemotherapy.

We conducted a retrospective review of electronic medical records in the database of the breast cancer registry of Chang Gung Memorial Hospital collected between January 2012 and December 2021, and identified 248 consecutive cN0 breast cancer patients without distant metastasis at initial presentation who underwent NAC. The study was approved by the Institutional Review Board of Chang Gung Memorial Hospital (IRB No: 202201113B0), and the requirement for written informed consent was waived. For all breast cancer patients who consider NAC at our institution, a multidisciplinary team routinely conducts a review for individual case assessment of the clinical breast cancer stage and makes recommendations for NAC regimens according to the institution’s treatment guidelines. Clinical nodal status was based on physical examination and ultrasonographic findings with fine-needle aspiration or core-needle biopsy of any suspicious lymph nodes. Patients with no suspicious lymph nodes on physical examination and ultrasound or a negative result confirmed by fine-needle aspiration or core-needle biopsy were defined as cN0. The assessment of distant metastases was mainly based on CT findings, including chest, abdomen, pelvis, and bone scans. Patients with clinical T4 disease (n = 6), synchronous contralateral breast cancer (n = 6), or a history of ipsilateral breast cancer (n = 7) at presentation, or no subsequent axillary surgery after NAC (n = 7) were excluded. The details regarding the data selection are shown in Figure 1. Finally, 222 eligible patients with clinical T1-3N0M0 primary invasive breast cancer were identified. Data on clinical information, including age, tumor histology, biologic subtype, tumor size, TNM stage, tumor grade, NAC regimens, operation type, and pathological findings on the final pathology of these cases were collected from the electronic database for analyses.

### 2.2. Tumor Biological Assessment before Neoadjuvant Chemotherapy

Tumor histology, grade, Ki67, estrogen receptor (ER), progesterone receptor (PR), and HER2 status of all patients were confirmed in specimens obtained by core needle biopsy before NAC. Grading of invasive cancers was performed according to the method of Elston and Ellis [28]. The cutoff point for Ki67 was 20%. Positive ER and PR statuses were defined as tumors with >1% positively nuclear-stained cells. HER2 status was evaluated using immunohistochemistry (IHC). The HER2 status was defined as negative when the IHC score was 0 or 1+. Fluorescence in situ hybridization (FISH) was mandatory in addition to IHC for those with a 2+ IHC score. An IHC score of 3 or HER2 amplification using FISH was defined as HER2 positive. An IHC score of ER or PR > 1% was defined as hormone receptor (HR) positive. In this study, patients were categorized into four biological subtypes based on combinations of HR and HER2 status: HR-positive/HER2-negative, HR-positive/HER2-positive, HR-negative/HER2-positive, and HR-negative/HER2-negative.

### 2.3. Axillary Ultrasound Procedures for Assessing the Axillary Nodal Status

In our institution, axillary ultrasound is routinely performed for axillary nodal staging of breast cancer patients before treatment initiation. Ultrasound features of a normal lymph node include an oval shape, a cortex of <2 mm, a cortex of uniform thickness, and a normal plump echogenic hilum. If any suspicious lymph nodes were observed during evaluation by axillary ultrasound, FNAC or core needle biopsy under ultrasound guidance was subsequently performed to obtain specimens for pathological examination.

### 2.4. Neoadjuvant Chemotherapy Regimens

Of the 91 patients with HR-positive/HER2-negative tumors, the majority (79/91, 86.8%) received NAC with an anthracycline-based regimen, followed by or combined with a taxane-based regimen. Of the remaining 12 patients, five were administered an anthracycline-based regimen only, and seven were administered a taxane-based regimen only. Among the 50 triple-negative breast cancer patients, all received sequential treatment with anthracycline-based and taxane-based regimens, and 37 received taxane-based concurrently with platinum-based regimens. All 81 patients with HER2-positive disease received anti-HER2 therapy in addition to chemotherapy, of which 47 received a single agent with hercetpin and 34 received dual blockade with herceptin and pertuzumab. Regarding chemotherapy for HER2-positive patients, 51 received an anthracycline-based regimen followed by a taxane-based regimen and 30 received a taxane-based regimen concurrently with a platinum-based regimen.

### 2.5. Subsequent Surgical Intervention

All patients underwent breast and axillary surgery 4–6 weeks after NAC completion. Within 4 weeks before surgery, all patients underwent breast ultrasonography and mammography for preoperative radiological evaluation. The extent of breast resection would be planned based on the original tumor size while the scattered distribution of the tumor response after NAC was observed; the extent of breast resection would be determined based on the new tumor size when the tumor was found to have a concentric response after NAC. BCS is indicated for tumors that are clinically assessed to be resectable with clear margins and acceptable cosmetic results, based on the location and size of the tumors. In general, mastectomy is indicated for multicentric tumors, diffuse suspicion of malignant microcalcifications, resection of larger tumors affecting cosmetic outcomes, persistently positive margins of excision, or patient preference. At our institution, the surgeon routinely performed ultrasound-guided BCS procedures. In each case undergoing BCS, intraoperative frozen section analysis of the cavity margin was performed to determine tumor resection margin status. If intraductal or invasive carcinoma was detected, re-excision of the involved margin would be carried out to achieve negative cavity margin status during the same operation. Therefore, all patients who received BCS in our series were finally confirmed to have negative margins. Regarding the reasons for 49 patients undergoing mastectomy, 13 were large tumors, 15 were diffuse suspicion of malignant microcalcifications, 8 were nipple involvement by tumor, 11 were patient preference, and 2 were persistently positive margins of excision. The SLN was routinely identified using the radiocolloid technique, with periareolar or peritumor injection of technetium 99m-labeled sulfur colloid, either in the morning or the day before surgery. Complementary ALND was performed in cases of SLN mapping failure or SLN metastasis proven by SLNB.

### 2.6. Pathological Evaluation of Surgical Specimens after Neoadjuvant Chemotherapy

All SLNs and breast specimens were fixed in formalin, embedded in paraffin, and examined using hematoxylin and eosin (H&E) stain. Each SLN was serially cut through the hilum at 2 mm intervals. If no evidence of carcinoma was detected by H&E staining for SLN analysis, further immunohistochemical examination for cytokeratin was performed. ypN0 was defined as the absence of tumor cells in the ALNs after NAC. Owing to the study’s purpose of identifying factors that predict the complete absence of tumors in ALNs and thus potentially avoid axillary surgery, we defined ypN0 using stricter criteria than those commonly used. The presence of any tumor cells in the ALNs, including isolated tumor cells (≤0.2 mm), micrometastases (>0.2 mm and ≤2 mm), or macrometastasis (>2 mm), was considered ypN+. Breast pCR was defined as the absence of residual invasive carcinoma in the breast after NAC (ypT0/is). Breast non-pCR was defined as a residual invasive carcinoma in the breast after NAC. The final pathological evaluation of the resected breast specimens included histological pattern, tumor grade, tumor response, tumor size, and presence of lymphovascular invasion (LVI).

### 2.7. Follow-Up

All patients are scheduled for regular visits every 3–6 months during the first 2 years, every 6 months years three to five, and annually thereafter. The follow-up examination methods include physical examination at each return visit and annual mammography with ultrasound. In symptomatic cases or when clinically indicated, chest X-ray, liver ultrasound, bone scan, or CT were carried out. Clinical outcome data were obtained from electronic medical records. Disease-free survival (DFS) was calculated from initial diagnosis until the occurrence of the first local, regional or distant recurrence.

### 2.8. Statistical Analysis

The correlation of baseline characteristics before NAC, including age, tumor size, clinical T category, tumor characteristics (histology type, tumor grade, biologic subtype, and Ki67 index), type of breast surgery, and histopathological findings of resected specimens, including the presence or absence of breast pCR and LVI with ALN metastasis, was analyzed using univariate and multivariate analyses. Analysis of risk factors for ALN metastasis in subgroups of patients who did not achieve breast pCR showed that all variables except breast pCR were the same as those used in the entire population. Categorical variables were compared using Pearson’s chi-squared test, and continuous variables were assessed using the t-test. Variables with *p* < 0.100 in the univariate analyses were entered into multivariate analysis using a logistic regression model. *p* value ≤ 0.05 was considered statistically significant. All statistical analyses were performed using the SPSS software (version 20.0; SPSS Inc., Chicago, IL, USA).

## 3. Results

### 3.1. Patient and Tumor Characteristics

The clinical and pathological characteristics of the patients in this study cohort before and after NAC are summarized in Table 1. All patients were female, and the median age of the patients was 46.5 (range, 24–71) years. The median tumor size was 3.3 (interquartile range (IQR): 1.5) cm. The most common histological tumor type was invasive ductal carcinoma (212/222, 95.5%). Most patients had a higher tumor grade at presentation, with 44.1% being grade 2 and 40.1% being grade 3. The most common clinical T category was T2 (187/222, 84.2%). The distribution of biologic subtypes in the 222 patients was as follows: 91 (41.0%) were HR-positive/HER2-negative, 51 (23.0%) were HR-positive/HER2-positive, 30 (13.5%) were HR-negative/HER2-positive, and 50 (22.5%) were HR-negative/HER2-negative. Regarding proliferation markers, high Ki67 levels (≥20) were observed in 132 patients (59.5%).

### 3.2. Breast and Axillary Surgery

Of the 222 patients in the cohort, 173 (77.9%) underwent BCS and 49 (22.1%) underwent mastectomy after NAC. All patients were scheduled for SLN mapping, and 215 were successful and seven failed. As a result, 201 patients received SLNB only, 7 patients underwent ALND due to the failure of SLN mapping, and the remaining 14 patients underwent subsequent ALND after SLN metastasis was confirmed. ALND was not performed in three patients with isolated tumor cells found in the SLN. The median number of SLNs removed was two (range, 1–7).

### 3.3. Pathological Findings of Tumor in Breast and Axilla

Overall, 40.5% (90/222) of the patients achieved pCR in the breast (ypT0/is). Of the 205 cases with successful SLNB, SLNs involvement was observed to be macrometastasis in 9 (4.4%), micrometastasis in 5 (2.4%), isolated tumor cells in 3 (1.5%), and no metastasis in 188 (91.7%) patients. Of the seven patients who underwent ALND due to SLN mapping failure, none had ALN metastasis. Of the 14 patients who underwent ALND following SLN metastasis, four were found to have lymph node metastasis in addition to SLNs. The overall rate of pathological node positivity after NAC (ypN+) was 7.7% (17/222). In the study cohort, LVI was absent in most cases (202/222, 91.0%).

### 3.4. Predictors of Axillary Lymph Node Status

A comparison between ypN+ and ypN0 patients is shown in Table 2. Univariate analysis showed that significant factors associated with ALN metastasis included tumor histologic type, tumor grade, biological subtype, Ki-67, the presence of LVI, and breast pCR. Compared with the patients in the ypN0 group, patients in the ypN+ group were more likely to have a non-ductal histologic type (17.6% vs. 3.4%; *p* = 0.032). Patients with a lower-grade tumor had a significantly higher rate of ALN metastasis than those with a higher-grade tumor (grade 1 vs. grade 2 vs. grade 3, 5/35 (14.3%) vs. 10/98 (10.2%) vs. 2/89 (2.2%); *p* = 0.034). ALN status varied by biological subtype with ypN+ disease being most common in HR-positive/HER2-negative (12/91, 13.2%) breast cancer followed by HR-negative/HER2-positive (2/30, 6.7%), HR-positive/HER2-positive (2/51, 3.9%), and HR-negative/HER2-negative (1/50, 2.0%) (*p* = 0.064). Tumors with a lower proliferation index were associated with higher ypN+ rates (Ki-67 < 20 vs. Ki-67 ≥ 20, 9/61 (14.8%) vs. 7/132 (5.3%); *p* = 0.047). The presence of LVI on the final pathology was more common in patients with ypN+ (58.8%) than in those with ypN0 (4.9%) (*p* < 0.0001). Patients with residual invasive carcinoma in the breast were more likely to have ALN metastasis than those with breast pCR (17/132 [12.9%] vs. 0/90 [0.0%]; *p* < 0.001). There were no significant differences in age (*p* = 0.783), tumor size (*p* = 0.953), and clinical T category (*p* = 0.486). In this cohort, all 90 patients with breast pCR had no evidence of ALN metastasis. In addition, in our study, it was observed that when the tumor had a high grade, high Ki-67 performance, and belonged to HR-negative/HER2-positive or HR-positive/HER2-positive biological subtypes, a breast pCR was more likely to be obtained after NAC (Table 3).

On multivariate analysis, the greatest increase in relative risk for ypN+ was seen in patients who had the presence of LVI on final pathologic findings (odds ratio [OR] = 29.366; 95% confidence interval [CI], 7.146–120.682; *p* < 0.0001). Tumor histologic type, tumor grade, biological subtype, and ki-67 were not independent risk predictors of ypN+. Although breast non-pCR as a risk predictor for ypN+ did not reach statistical significance in the multivariate analysis, it still showed a trend of correlation (OR = 13.896; 95% CI, 0.752–256.837; *p* = 0.077). Furthermore, according to the result that all patients in this study cohort had a negative ALN status as long as they achieved breast pCR after NAC, we further conducted univariate and multivariate analyses of risk predictors associated with ypN+ among patients who did not achieve breast pCR.

### 3.5. Risk Predictors of Axillary Lymph Node Metastasis among Breast Non-pCR Patients

The clinical and pathological characteristics of the patients with breast non-pCR are shown in Table S1. A comparison of ypN+ and ypN0 among breast non-pCR patients is shown in Table 4. On univariate analysis, we found that breast surgery type and LVI were significant factors associated with ALN status. Mastectomies were performed more frequently in patients with ypN+ (52.9%) than in those with ypN0 (20.9%) (*p* = 0.013). The presence of LVI on the final pathology was more common in patients with ypN+ (58.8%) than in those with ypN0 (7.0%) (*p* < 0.0001). There were no significant differences in age (*p* = 0.879), tumor size (*p* = 0.699), tumor histology (*p* = 0.092), clinical T category (*p* = 0.512), tumor grade (*p* = 0.333), biological subtype (*p* = 0.353), and Ki-67 (*p* = 0.481). Multivariate analysis demonstrated that mastectomy breast surgery (OR = 3.420; 95% CI, 1.004–11.648; *p* = 0.049) and the presence of LVI (OR = 16.927; 95% CI, 4.882–58.687; *p* < 0.0001) were independent risk predictors of ALN metastasis.

**Figure 2:**
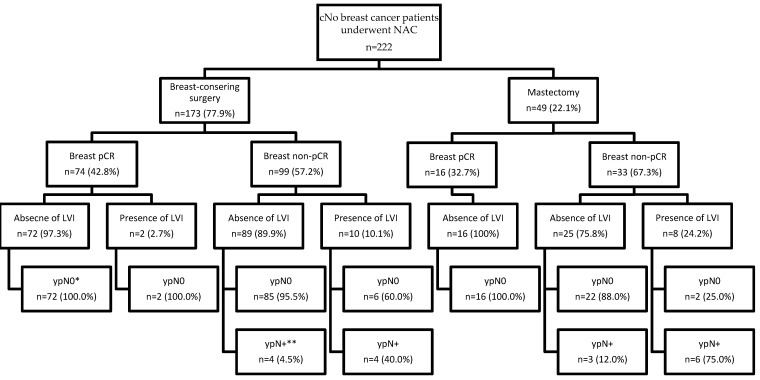
Results of axillary lymph node status for patients with clinically node-negative breast cancer after neoadjuvant chemotherapy stratified by different surgical procedures, tumor response in the breast, and presence or absence of LVI. cN0, clinically node-negative; NAC, neoadjuvant chemotherapy; pCR, pathologic complete response; LVI, lymphovascular invasion. Breast pCR was defined as residual invasive carcinoma in the breast after neoadjuvant chemotherapy. * ypN0 was defined as no tumor cells in the axillary lymph nodes after neoadjuvant chemotherapy. ** ypN+ was defined as the presence of any tumor cells in the axillary lymph nodes after neoadjuvant chemotherapy, including isolated tumor cells (≤0.2 mm), micrometastases (>0.2 mm and ≤2 mm), or macrometastasis (>2 mm).

The results of axillary lymph node status stratified by the type of breast surgery, achievement of breast pCR, and presence of LVI are shown in Figure 2. For patients with breast pCR, the incidence of yN0 was 100%, regardless of the surgical procedure and the presence or absence of LVI. In the subgroups of patients who underwent BCS and demonstrated breast non-pCR, the incidence of ypN+ was as low as 4.5% in the absence of LVI and as high as 40% in the presence of LVI. In subgroups of patients who underwent mastectomy and did not achieve breast pCR, the incidence of ypN+ was 12% in the absence of LVI and as high as 75% in the presence of LVI.

### 3.6. Clinical Outcome

The median follow-up time was 42.1 months (range 5.2–118.47 months) and the last follow-up date was 30 April 2022. A total of 10 (4.5%) patients had recurrent disease. The median DFS was 41.2 months. The time interval from diagnosis to recurrence in patients with recurrent disease ranged from a minimum of 33 months to a maximum of 882 months (Table S2). Regarding recurrence pattern, two patients (0.9%) had only ipsilateral breast tumor recurrence, one patient (0.5%) had only regional recurrence, six patients (2.7%) had only distant recurrence, and one patient (0.5%) had simultaneous regional and distant recurrence. All 10 patients with recurrent disease did not achieve breast pCR after NAC, three of them had the presence of LVI, and the remaining seven did not. There were two deaths due to recurrent disease.

## 4. Discussion

To the best of our knowledge, there are still no reliable preoperative imaging modalities that can accurately predict the presence of residual breast tumors and the true status of ALN in patients with breast cancer after NAC. As a result, current surgical treatment guidelines have not changed the basic concept of invasive surgery, including the breast and axilla, although their recommended procedures vary according to individual preoperative assessment of tumor response. In the absence of preoperative assessment tools comparable to pathological examination, surgery for primary lesions in the breast after NAC not only provides the tumor response to the drugs, and thus affects the choice of subsequent adjuvant therapy, but also achieves the purpose of local treatment. However, unlike breast surgery, the clinical significance of axillary surgery for breast cancer classified as cN0, including the absence of lymph node metastases or low-volume occult lymph node metastases, is primarily to provide information for the determination of clinically relevant ALN status. Furthermore, the administration of NAC is known to eradicate lymph node metastases [29,30], and a significantly higher pCR rate in ALNs than in the breast has been observed in cN+ patients receiving NAC [26,31,32,33]. Therefore, it is logical to infer the lymph node status of cN0 breast cancer based on the biological characteristics of the tumor and its response to drugs. In the current study, all the parameters used for analysis were routine tumor-related examinations of breast cancer before and after NAC; hence, the derived results were closer to general clinical experience and provide a clinical reference. The results of our study demonstrated that in patients with cN0 breast cancer following NAC, the accompanying ALN status was negative, as long as the pCR of the breast was confirmed. For those who did not achieve breast pCR, the likelihood of a positive ALN status was significantly higher in the presence of LVI. Furthermore, the factors identified in the current study would be useful for providing evidence regarding stratification in developing treatment strategies.

Differences in the way subjects clinically defined cN0 and the biological subtypes included in subjects, the proportions achieving breast pCR varied widely for the overall ypN+ rates observed in each cN0 breast cancer patient undergoing NAC [25,26,27,34,35,36,37,38]. Murphy et al. reported a ypN+ rate of 22% in patients registered in the National Cancer Database, without knowing whether axillary ultrasound was routinely performed [34]. van der Noordaa et al. observed a ypN+ rate of 14.5% with routine axillary ultrasound combined with fine-needle aspiration of suspected lymph nodes [35]. A lower ypN+ rate of only 3.4% was observed in a prospective cohort study by Tadros et al. in the cN0 subgroup that included only triple-negative and HER2-positive breast cancers [26]. In a study conducted by Ryu et al. using the Korean Breast Cancer Society Registry database, all patients with cT1-3N0 were found to have a breast pCR rate of 19.7% and a ypN+ rate of 16.7% regardless of the biological subtype [38]. In our study, the low ypN+ rate of 7.7% could be explained by routine axillary ultrasound combined with ultrasound-guided fine needle aspiration or core needle biopsy to confirm cN0 status; nearly 60% of patients had triple-negative or HER2-positive breast cancer and all populations had a high probability of achieving breast pCR of 40.5% regardless of biological subtype.

Several studies have demonstrated a strong correlation between breast pCR and ALN negativity [26,27,35,36,37,38]. Extremely low ypN+ rates were consistently observed in a cohort of cN0 breast cancer patients with HER2-positive or triple-negative breast cancer who achieved breast pCR after NAC. In a study by Barron et al., both HER2-positive and triple-negative subtypes had an ALN metastasis rate of 1.6% when breast pCR was achieved [27]. Similar results were obtained in a study involving 442 cT1-3 and cN0 breast cancer patients receiving NAC, with ypN+ rates of 0.9% for HER2-positive and 1.5% for triple-negative [37]. In a prospective study from MD Anderson, HER2-positive and triple-negative subtypes were found to be completely free of ALN metastasis in the context of confirmed breast pCR [26], which is consistent with our findings. As for the ypN+ rate of the HR-postive/HER2-negative subtype, some related studies have shown that it is slightly higher than that of HER-2 and triple-negative subtypes, but this is still controversial. Barron et al. and Samiei et al. observed that the ypN+ rate of HR-postive/HER2-negative subtype was 4.5% and 6.7%, respectively [27,37]; in contrast, Ryu et al. found that the ypN+ rate in the same subtype population was 0% [38]. In a retrospective study by Ryu et al., there were 647 cases of cT1-3 and cN0 breast cancer with HR-postive/HER2-negative subtype, of which 96 cases with breast pCR were confirmed to have no ALN metastasis, which is consistent with our findings [38]. However, the inconsistency in results on HR-positive/HER2-negative subtype breast cancer could be caused by the lack of prospective studies so far. In the future, prospective studies on this subtype are needed to resolve this controversy.

To investigate the predictive value of breast pCR for negative ALN status after NAC in patients with cN0 breast cancer, we performed a meta-analysis of recently published articles. Articles included in the analysis meet the clinical T category of the research subjects below T3 and the postoperative ALN status data of different biologic subtypes in patients with cN0 after NAC. The baseline characteristics of studies included in this meta-analysis of breast pCR and ypN0 are summarized in Table S3. The results after summing the individual biologic subtypes from the different studies and the data from our study showed that the highest prevalence of ypN0 was observed in HR-negative/HER2-positive (2353/2487, 94.6%), followed by HR-negative/HER2-negative (6567/7263, 90.4%), HR-positive/HER2-positive (4565/5194, 87.9%) and HR-positive/HER2-negative (4872/6793, 71.7%). The sensitivity of using breast pCR as a diagnostic test to identify ypN0 in different biological subtypes of cN0 breast cancer patients receiving NAC is very high, ranging from 96.3% for HR-positive/HER2-negative to 99.1% for HR-negative/HER2-positive. The specificity of breast pCR in diagnosing ypN0 status is generally low regardless of biological subtype, ranging from 14.0% for HR-negative/HER2-negative to 31.8% for HR-positive/HER2-negative (Table S4). The forest plots for these results show a strong relationship between breast pCR and ypN0 status (Figure S1). The pooled OR was 11.09 (95% CI, 7.81–15.75) in HR-positive/HER2-negative subgroup, 9.86 (95% CI, 7.15–13.60) in HR-positive/HER2-positive subgroup, 12.29 (95% CI, 7.12–21.23) in HR-negative/HER2-positive subgroup, 8.92 (95% CI, 6.47–12.30) in HR-negative/HER2-negative subgroup and 14.71 (95% CI, 12.29–17.61) in all patients regardless of biological subtype.

In the current study, the presence of LVI was confirmed as a risk predictor for ALN metastasis in both the entire population and the breast non-pCR subpopulation. Theoretically, the process of lymphatic system metastasis is step-by-step, and LVI is considered a crucial step in breast cancer metastasis [39,40]. However, most studies on ALN metastasis after NAC in cN0 breast cancer did not include LVI as a factor in the analysis; therefore, LVI has not been identified as a risk factor for ALN metastasis in the previous studies. In contrast, the presence of LVI has been shown to be highly associated with ALN metastases in many studies investigating the risk factors for ALN metastasis in patients with cN0 breast cancer undergoing primary surgery [41,42,43]. In a study of 400 patients with cT1-2 and cN0 breast cancers who received SLNB, patients with LVI (presence of LVI, 51.3% vs. absence of LVI, 30.3%; OR = 2.07, 95% CI, 1.34–3.19) were more likely to have positive SLNs [42]. Gajdos et al. reported a retrospective study of 850 cases showing that LVI was highly associated with ALN metastases, with LN positivity confirmed in 51% of patients with LVI compared to 19% of patients without LVI [43]. In the present study, there was a significant association between the presence of LVI and ALN metastases, showing that patients with LVI were more likely to have positive ALNs than those without LVI in the overall population (presence of LVI, 50% vs. absence of LVI, 3.5%; OR = 29.366, 95% CI, 7.146–120.682), which was consistent with the research findings mentioned above. Similar results were observed in the breast non-pCR subgroups, with patients with LVI having higher ypN+ rates than those without LVI (presence of LVI, 55.6% vs. absence of LVI, 6.1%; OR = 19.107, 95% CI, 5.733–63.676).

In the present study, the type of breast surgery was identified to be associated with the likelihood of ypN+ in patients with breast non-pCR subgroups. Patients who underwent total mastectomy had higher rates of ypN+ than those who underwent BCS (mastectomy, 27.2% vs. BCS, 8.1%; OR = 3.420, 95% CI, 1.004–11.648). The selection process for the type of breast surgery after NAC is complex and usually needs to be developed through multidisciplinary team planning while respecting patient preferences. A possible explanation for the association between breast surgery type and ALN metastasis in the subgroup of patients with breast non-pCR is that, in comparison with the cases of mastectomy, the patients who planned to undergo BCS after NAC were more likely to have smaller residual breast disease, which indirectly indicates a lower possibility of ALN metastasis.

The two main factors associated with ALN status identified in our study, breast pCR as a predictor of ALN negativity and the presence of LVI as a predictor of ALN positivity, were only confirmed from the pathology of postoperative specimens. For patients with cN0 breast cancer after NAC, the current surgical treatment algorithm for preoperatively diagnosed ductal carcinoma in situ should be referred to despite the essential differences between the two diseases. That is, for patients whose preoperative assessment significantly did not achieve a breast pCR or those who are expected to undergo mastectomy, SLNB should still be performed alongside breast surgery according to the current treatment guidelines. On the other hand, for patients who are likely to achieve a breast pCR at preoperative evaluation and are planning to undergo BCS, SLNB should not be performed in the same surgery as BCS, but after final pathological confirmation of breast non-pCR. However, this idea needs to be confirmed in future prospective studies before clinical practice.

The use of image-guided minimally invasive breast biopsy to obtain pathological tissue to identify whether a breast lesion achieves pCR after NAC should be theoretically feasible, but the results in the published literature have varied widely [44,45,46]. The greatest difference between the positive and negative results of these studies is that the vacuum-assisted device system using the larger needle yields more; therefore, more pathologically representative tissue is obtained than the core needle biopsy. Other factors affecting the results are the choice of imaging tools suitable for the identification of the location and size of residual lesions, and the number of obtained pathological representations of the tumor. Once the ideal patient selection criteria and minimally invasive standard procedures are developed after overcoming the above factors, it may be possible to eliminate the need for breast surgery to confirm breast pCR and even omit axillary surgery.

The current study had several limitations. First, as this was a retrospective analysis conducted at a single institution, a selection bias may be unavoidable. Although the NAC regimens used in individual cases of different biological subtypes of tumors are different, each case has a multidisciplinary team discussion to formulate the choice of drugs, and the treatment responses of different biological subtypes are expected to be normally distributed. Second, we do not know whether the results of using ultrasound as an imaging diagnostic tool to confirm a patient’s ALN status as cN0 will change when other more sensitive diagnostic tools, such as FDG-PET/CT, are added. However, considering the popularity, facility, and financial burden of ultrasound, it is clinically more feasible as a diagnostic tool. Finally, since the vast majority of patients underwent SLNB without ALND in our study, we were unable to investigate how many of them were false-negative cases. However, the false-negative rate in our study was inferred to be acceptable based on current guidelines for patients with cN0 breast cancer and the low treatment failure rate of clinical outcomes during follow-up.

## 5. Conclusions

Our study shows an extremely high positive correlation between pCR status in the breast and negative ALN status in patients with cN0 breast cancer after NAC, regardless of the biological subtype, which supports the possibility of omitting axillary surgery if breast pCR is achieved. In contrast, regardless of the biological subtype of breast cancer, the presence of LVI in the breast is highly correlated with ALN positivity. It is clinically feasible to adopt a two-stage surgical plan for BCS in patients who are likely to achieve breast pCR before surgery. The plan is to first perform breast surgery, followed by axillary surgery for axillary staging once breast non-pCR is found. However, such surgical planning processes must be confirmed in prospective clinical trials before clinical application.

## Figures and Tables

**Table 1:** Clinical and tumor characteristics of the whole patient cohort after NAC.

Variable	No. of Cases	%
Age (years), median (IQR)	46.5 (13)	
<40	56	25.2
41–50	90	40.5
51–60	51	23.0
>60	25	11.3
Tumor size (cm), median (IQR)	3.3 (1.5)	
Tumor histology		
IDC	212	95.5
Others	10	4.5
Clinical T category ^a^		
T1	14	6.3
T2	187	84.2
T3	21	9.5
SBR grade ^b^		
1	35	15.8
2	98	44.1
3	89	40.1
Biologic subtype		
HR-positive/HER2-negative	91	41.0
HR-positive/HER2-positive	51	23.0
HR-negative/HER2-positive	30	13.5
HR-negative/HER2-negative	50	22.5
Ki-67		
<20	61	27.5
≥20	132	59.5
Unknown	29	13.0
Breast surgery type		
Mastectomy	49	22.1
Breast-conserving surgery	173	77.9
Lymphovascular invasion		
Present	20	9.0
Absent	202	91.0
Pathological breast status after NAC		
Breast non-pCR ^c^	132	59.5
Breast pCR ^d^	90	40.5
Pathological node status after NAC		
ypN+ ^e^	17	7.7
ypN0 ^f^	205	92.3

Abbreviations: NAC, neoadjuvant chemotherapy; IQR, interquartile range; IDC, invasive ductal carcinoma; HR, hormone receptor; HER2, human epidermal growth factor receptor 2; pCR, pathologic complete response. Figures are numbers with percentages in parentheses, unless otherwise stated. *^a^* T stage was defined according to the American Joint Committee on Cancer TNM classification. ^b^ Scarff Bloom Richardson grading system modified by Elston and Ellis. ^c^ Breast non-pCR was defined as a residual invasive carcinoma in the breast after NAC. ^d^ Breast pCR was defined as the absence of residual invasive carcinoma in the breast after NAC (ypT0/is). ^e^ ypN+ was defined as any tumor cells in the axillary lymph nodes after NAC, including isolated tumor cells (≤0.2 mm), micrometastases (>0.2 mm and ≤2 mm), or macrometastasis (>2 mm). ^f^ ypN0 was defined as the absence of tumor cells in the axillary lymph nodes after NAC.

**Table 2:** Univariate and multivariate analysis of factors influencing the lymph node status in the whole patient cohort after NAC.

Variable	Pathological Node Status after NAC		*p* Value	Multivariate Analysis *		
	ypN0 ^a^ (n = 205)	ypN+ ^b^ (n = 17)		Odds Ratio	95% CI	*p* Value
Age (years), median (IQR)	46 (14)	48 (8)	0.783			
≤40	53 (25.9)	3 (17.6)	0.661	-		
41–50	81 (39.5)	9 (52.9)				
51–60	47 (22.9)	4 (23.5)				
>60	24 (11.7)	1 (5.9)				
Tumor size (cm), median (IQR)	3.3 (1.5)	3.2 (1.3)	0.953	-		
Tumor histology			0.032			
IDC	198 (96.6)	14 (82.4)		Reference		
Others	7 (3.4)	3 (17.6)		3.410	0.568–20.476	0.180
Clinical T category ^c^			0.486	-		
T1	13 (6.3)	1 (5.9)				
T2	174 (84.9)	13 (76.5)				
T3	18 (8.8)	3 (17.6)				
SBR grade ^d^			0.034			
1	30 (14.6)	5 (29.4)		Reference		
2	88 (42.9)	10 (58.8)		0.534	0.126–2.261	0.394
3	87 (42.4)	2 (11.8)		0.196	0.028–1.378	0.101
Biologic subtype			0.064			
HR-positive/HER2-negative	79 (38.5)	12 (70.6)		Reference		
HR-positive/HER2-positive	49 (23.9)	2 (11.8)		0.670	0.134–3.365	0.627
HR-negative/HER2-positive	28 (13.7)	2 (11.8)		0.575	0.093–3.556	0.552
HR-negative/HER2-negative	49 (23.9)	1 (5.8)		0.795	0.115–5.474	0.816
Ki-67			0.047			
<20	52 (25.4)	9 (52.9)		Reference		
≥20	125 (61.0)	7 (41.2)		0.995	0.252–3.926	0.995
Unknown	28 (13.7)	1 (5.9)		0.308	0.042–.241	0.245
Lymphovascular invasion			<0.0001			<0.0001
Present	10 (4.9)	10 (58.8)		29.366	7.146–120.682	
Absent	195 (95.1)	7 (41.2)		Reference		
Pathological breast status after NAC			<0.001			0.077
Breast non-pCR ^e^	115 (56.1)	17 (100.0)		13.896	0.752–256.837	
Breast pCR ^f^	90 (43.9)	0		Reference		

Abbreviations: NAC, neoadjuvant chemotherapy; IQR, interquartile range; CI, confidence interval; IDC, invasive ductal carcinoma; HR, hormone receptor; HER2, human epidermal growth factor receptor 2; pCR, pathologic complete response. Figures are numbers with percentages in parentheses, unless otherwise stated. Figures are numbers with percentages in parentheses, unless otherwise stated. * By using the Bayesian generalized linear models for modeling rare events data. ^a^ ypN0 was defined as the absence of tumor cells in the axillary lymph nodes after NAC. ^b^ ypN+ was defined as any tumor cells in the axillary lymph nodes after NAC, including isolated tumor cells (≤0.2 mm), micrometastases (>0.2 mm and ≤2 mm), or macrometastasis (>2 mm). ^c^ T stage was defined according to the American Joint Committee on Cancer TNM classification. ^d^ Scarff Bloom Richardson grading system modified by Elston and Ellis. ^e^ Breast non-pCR was defined as a residual invasive carcinoma in the breast after NAC. ^f^ Breast pCR was defined as the absence of residual invasive carcinoma in the breast after NAC (ypT0/is).

**Table 3:** Univariate and multivariate analysis of factors associated with breast pathologic complete response in the whole patient cohort after NAC.

Variable	Pathological Breast Status		*p* Value	Multivariate Analysis *		
	Breast Non-pCR ^a^ (n = 132)	Breast pCR ^b^ (n = 90)		Odds Ratio	95% CI	*p* Value
Age (years), median (IQR)	47 (10)	46 (18)	0.664			
≤40	29 (22.0)	27 (30.0)	0.127	-		
41–50	62 (47.0)	28 (31.1)				
51–60	28 (21.2)	23 (25.6)				
>60	13 (9.8)	12 (13.3)				
Tumor size (cm), median (IQR)	3.3 (1.3)	3.2 (1.5)	0.314			
Tumor histology			0.052			
IDC	123 (93.2)	89 (98.9)		Reference		
Others	9 (6.8)	1 (1.1)		0.541	0.050–5.821	0.612
Clinical T category ^c^			0.896	-		
T1	9 (6.8)	5 (5.6)				
T2	110 (83.3)	77 (85.6)				
T3	13 (9.8)	8 (8.9)				
SBR Grade ^d^			<0.0001			
1	31 (23.5)	4 (4.4)		Reference		
2	66 (50.0)	32 (35.6)		2.519	0.748–8.484	0.136
3	35 (26.5)	54 (60.0)		5.624	1.546–20.456	0.009
Biologic subtype			<0.0001			
HR-positive/HER2-negative	71 (53.8)	20 (22.2)		Reference		
HR-positive/HER2-positive	27 (20.5)	24 (26.7)		2.533	1.138–5.637	0.023
HR-negative/HER2-positive	12 (9.1)	18 (20.0)		2.923	1.132–7.546	0.027
HR-negative/HER2-negative	22 (16.7)	28 (31.1)		2.164	0.946–4.949	0.067
Ki-67			<0.0001			
<20	53 (40.2)	8 (8.9)		Reference		
≥20	65 (49.2)	67 (74.4)		2.682	1.053–6.829	0.039
Unknown	14 (10.6)	15 (16.7)		4.354	1.399–13.554	0.011
Operation type			0.203			
Mastectomy	33 (25.0)	16 (17.8)				
Breast-conserving surgery	99 (75.0)	74 (82.2)				

Abbreviations: NAC, neoadjuvant chemotherapy; pCR, pathologic complete response; CI, confidence interval; IQR, interquartile range; HR, hormone receptor; HER2, human epidermal growth factor receptor 2. Figures are numbers with percentages in parentheses, unless otherwise stated. Figures are numbers with percentages in parentheses, unless otherwise stated. * By using the logistic regression model. ^a^ Breast non-pCR was defined as a residual invasive carcinoma in the breast after NAC. ^b^ Breast pCR was defined as the absence of residual invasive carcinoma in the breast after NAC (ypT0/is). ^c^ T stage was defined according to the American Joint Committee on Cancer TNM classification. ^d^ Scarff Bloom Richardson grading system modified by Elston and Ellis.

**Table 4:** Univariate and multivariate analysis of factors influencing the lymph node status in the subgroup patients with breast non-pCR after NAC.

Variable	Pathological Node Status after NAC		*p* Value	Multivariate Analysis *		
	ypN0 ^a^ (n =115)	ypN+ ^b^ (n = 17)		Odds Ratio	95% CI	*p* Value
Age (years), median (IQR)	47 (12)	48 (8)	0.879			
≤40	26 (22.6)	3 (17.6)		-		
41–50	53 (46.1)	9 (52.9)				
51–60	24 (20.9)	4 (23.5)				
>60	12 (10.4)	1 (5.9)				
Tumor size (cm), median (IQR)	3.3 (1.3)	3.2 (1.3)	0.699	-		
Tumor histology			0.092	-		
IDC	109 (94.8)	14 (82.4)				
Others	6 (5.2)	3 (17.6)				
Clinical T category ^c^			0.512	-		
T1	8 (7.0)	1 (5.9)				
T2	97 (84.3)	13 (76.5)				
T3	10 98.7)	3 (17.6)				
SBR grade ^d^			0.333	-		
1	26 (22.6)	5 (29.4)				
2	56 (48.7)	10 (58.8)				
3	33 (28.7)	2 (11.8)				
Biologic subtype			0.353	-		
HR-positive/HER2-negative	59 (51.3)	12 (70.6)				
HR-positive/HER2-positive	25 (21.7)	2 (11.8)				
HR-negative/HER2-positive	10 (8.7)	2 (11.8)				
HR-negative/HER2-negative	21 (18.3)	1 (5.9)				
Ki-67			0.481	-		
<20	44 (38.3)	9 (52.9)				
≥20	58 (50.4)	7 (41.2)				
Unknown	13 (11.3)	1 (5.9)				
Breast surgery type			0.013			
Mastectomy	24 (20.9)	9 (52.9)		3.420	1.004–11.648	0.049
Breast-conserving surgery	91 (79.1)	8 (47.1)		Reference		
Lymphovascular invasion			<0.0001			<0.0001
Present	8 (7.0)	10 (58.8)		16.927	4.882–58.687	
Absent	107 (93.0)	7 (41.2)		Reference		

Abbreviations: pCR, pathologic complete response; NAC, neoadjuvant chemotherapy; CI, confidence interval; IQR, interquartile range; IDC, invasive ductal carcinoma; HR, hormone receptor; HER2, human epidermal growth factor receptor 2. Figures are numbers with percentages in parentheses, unless otherwise stated. * By using the logistic regression model. *^a^* ypN0 was defined as the absence of tumor cells in the axillary lymph nodes after NAC. ^b^ ypN+ was defined as any tumor cells in the axillary lymph nodes after NAC, including isolated tumor cells (≤0.2 mm), micrometastases (>0.2 mm and ≤2 mm), or macrometastasis (>2 mm). ^c^ T stage was defined according to the American Joint Committee on Cancer TNM classification. ^d^ Scarff Bloom Richardson grading system modified by Elston and Ellis.

## Data Availability

The data presented in this study are available on request from the corresponding author. The data are not publicly available due to ethical regulations.

## Supplementary Materials

The following supporting information can be downloaded at: https://www.mdpi.com/article/10.3390/cancers14184451/s1, Table S1: Clinical and tumor characteristics of the patients with breast non-pCR after NAC; Table S2: Summary of clinical and pathologic features of patients with recurrent disease; Table S3: Baseline characteristics of studies included in this meta-analysis of breast pCR and ypN0^a^; Table S4: Using breast pCR as a diagnostic test in different biological subtypes for identifying ypN0 in cN0 breast cancer patients undergoing NAC after summing the results of studies included in the meta-analysis. Figure S1: Meta-analysis forest plots display the estimated odds ratio impact of breast pCR on predicting the ypN0 results from a group of studies plus a summary measure in different biologic subtype. HR, hormone receptor; HER2, human epidermal growth factor receptor 2.
