# Supplementary material for: Factors Associated with Axillary Lymph Node Status in Clinically Node-Negative Breast Cancer Patients Undergoing Neoadjuvant Chemotherapy
Source: Cancers (Basel). 2022 Sep 14;14(18):4451. doi: 10.3390/cancers14184451 (PMC9497171; doi:10.3390/cancers14184451)
Supplement: Supplementary file 1 [file cancers-14-04451-s001.zip › cancers-1867062-supplementary.pdf]

Table S1. Clinical and tumor characteristics of the patients with breast non-pCR after NAC.

Variable	No. of cases	%
Age (years), median (IQR)		
<40	24	18.2
40-55	84	63.6
>55	24	18.2
Tumor size (cm), median (IQR)	3.3 (1.5)	
Tumor histology		
IDC	123	93.2
Others	9	6.8
Clinical T category[a]		
T1	9	6.8
T2	110	83.4
T3	13	9.8
SBR Grade[b]		
1	31	23.5
2	66	50.0
3	35	26.5
Biologic subtype		
HR-positive/HER2-negative	71	53.8
HR-positive/HER2-positive	27	20.5
HR-negative/HER2-positive	12	9.0
HR-negative/HER2-negative	22	16.7
Ki-67		
<20	53	40.2
≥20	65	49.2
Unknown	14	10.6
Breast surgery type		
Mastectomy	33	25.0
Breast-conserving surgery	99	75.0
Lymphovascular invasion		
Present	18	13.6
Absent	114	86.4
Pathological node status after NAC		

ypN+[c]	17	12.9
ypN0[d]	115	87.1

Abbreviations: pCR, pathologic complete response; NAC, neoadjuvant chemotherapy; IQR, interquartile range; IDC, invasive ductal carcinoma; HR, hormone receptor; HER2, human epidermal growth factor receptor 2. Figures are numbers with percentages in parentheses, unless otherwise stated.[a] T stage was defined according to the American Joint Committee on Cancer TNM classification. [b] Scarff Bloom Richardson grading system modified by Elston & Ellis. [c] ypN+ was defined as any tumor cells in the axillary lymph nodes after NAC, including isolated tumor cells (≤ 0.2 mm), micrometastases (> 0.2 mm and ≤ 2 mm), or macrometastasis (> 2 mm). [d] ypN0 was defined as the absence of tumor cells in the axillary lymph nodes after NAC.

Table S2. Summary of clinical and pathologic features of patients with recurrent disease.

Age (years)	Tumor histology	Tumor size (cm)	SBR grade	Biologic subtype	Breast surgery type	LVI	Pathological status after NAC	Time interval from diagnosis to recurrence (months)	Recurrences ite
32	IDC	2.6	2	HR-positive/ HER2-negative	BCS	Present	ypT2N1miM0	53	Ipsilateral Rotter's LNs
51	IDC	4.0	2	HR-positive/ HER2-negative	Mast.	Present	ypT1micN1M0	882	Ipsilateral neck LNs
47	IDC	3.0	1	HR-positive/ HER2-negative	BCS	Absent	ypT1cN0M0	89	Liver
43	IDC	4.5	2	HR-positive/ HER2-negative	Mast.	Absent	ypT2N0M0	60	Bone
52	IDC	2.1	2	HR-positive/ HER2-negative	BCS	Absent	ypT1bN0M0	87	Para-aortic LNs
43	IDC	4.8	2	HR-negative/ HER2-negative	BCS	Present	ypT2N0M0	41	Liver
43	IDC	2.4	2	HR-positive/ HER2-positive	BCS	Absent	ypT1cN0M0	33	Lung, liver, bone, ipsilateral axillary LNs
35	IDC	3.4	2	HR-negative/ HER2-positive	BCS	Absent	ypT1cN0M0	38	Liver
47	IDC	3.5	2	HR-positive/ HER2-negative	BCS	Absent	ypT1cN0M0	39	IBTR
39	IDC	4.5	2	HR-positive/ HER2-positive	BCS	Absent	ypT2N0M0	36	IBTR

Abbreviations: LVI, lymphovascular invasion; NAC, neoadjuvant chemotherapy; IDC, invasive ductal carcinoma; HR, hormone receptor; HER2, human epidermal growth factor receptor 2; BCS, breast-conserving surgery; Mast., mastectomy; LNs, lymph nodes; IBTR, ipsilateral breast tumor recurrence.

Table S3. Baseline characteristics of studies included in this meta-analysis of breast pCR and ypN0[a].

Study	Location	Sample size	Clinical stage	Classification of biologic subtype
Tadros et al.,2017 [26]	USA	290	cT1-2N0	HER2-positive<br>HR-negative/HER2-negative
Barron et al.,2018 [27]	USA	18093	cT1-2N0	HR-positive/HER2-negative<br>HR-positive/HER2-positive<br>HR-negative/HER2-positive<br>HR-negative/HER2-negative
Choi et al.,2019 [36]	Korea	200	cT1-3N0	HR-positive/HER2-negative<br>HR-positive/HER2-positive<br>HR-negative/HER2-positive<br>HR-negative/HER2-negative
Samiei et al.,2020 [37]	Netherlands	1674	cT1-3N0	ER-positive/HER2-negative ER-positive/HER2-positive<br>ER-negative/HER2-positive<br>ER-negative/HER2-negative
Ryu et al.,2022 [38]	Korea	1390	cT1-3N0	HR-positive/HER2-negative<br>HR-positive/HER2-positive<br>HR-negative/HER2-positive<br>HR-negative/HER2-negative
Yu et al.,2022	Taiwan	222	cT1-3N0	HR-positive/HER2-negative<br>HR-positive/HER2-positive<br>HR-negative/HER2-positive<br>HR-negative/HER2-negative

Abbreviations: pCR, pathologic complete response; HER2, human epidermal growth factor receptor 2; HR, hormone receptor; ER, estrogen receptor. [a] ypN0 was defined as the absence of tumor cells in the axillary lymph nodes after neoadjuvant chemotherapy.

Table S4. Using breast pCR as a diagnostic test in different biological subtypes for identifying ypN0 in cN0 breast cancer patients undergoing NAC after summing the results of studies included in the meta-analysis.

	Prevalence of ypN0	Sensitivity	Secificity
HR-positive/HER2-negative	71.7% (4872/6793)	96.3% (827/859)	31.8% (1889/5934)
HR-positive/HER2-positive	87.9% (4565/5194)	97.9% (1905/1946)	18.1% (588/3248)
HR-negative/HER2-positive	94.6% (2353/2487)	99.1%(1461/1474)	11.9% (121/1013)
HR-negative/HER2-negative	90.4% (6567/7263)	98.5%(40/2585)	14.0% (656/4678)
All patients	84.5% (18488/21869)	98.2%(6790/6916)	21.8% (3255/14953)

Abbreviations: pCR, pathologic complete response; cN0, clinically node-negative; NAC, neoadjuvant chemotherapy. [a] ypN0 was defined as the absence of tumor cells in the axillary lymph nodes after neoadjuvant chemotherapy.
