# Supplementary figures and images for: Factors Associated with Axillary Lymph Node Status in Clinically Node-Negative Breast Cancer Patients Undergoing Neoadjuvant Chemotherapy
Source: Cancers (Basel). 2022 Sep 14;14(18):4451. doi: 10.3390/cancers14184451 (PMC9497171; doi:10.3390/cancers14184451)

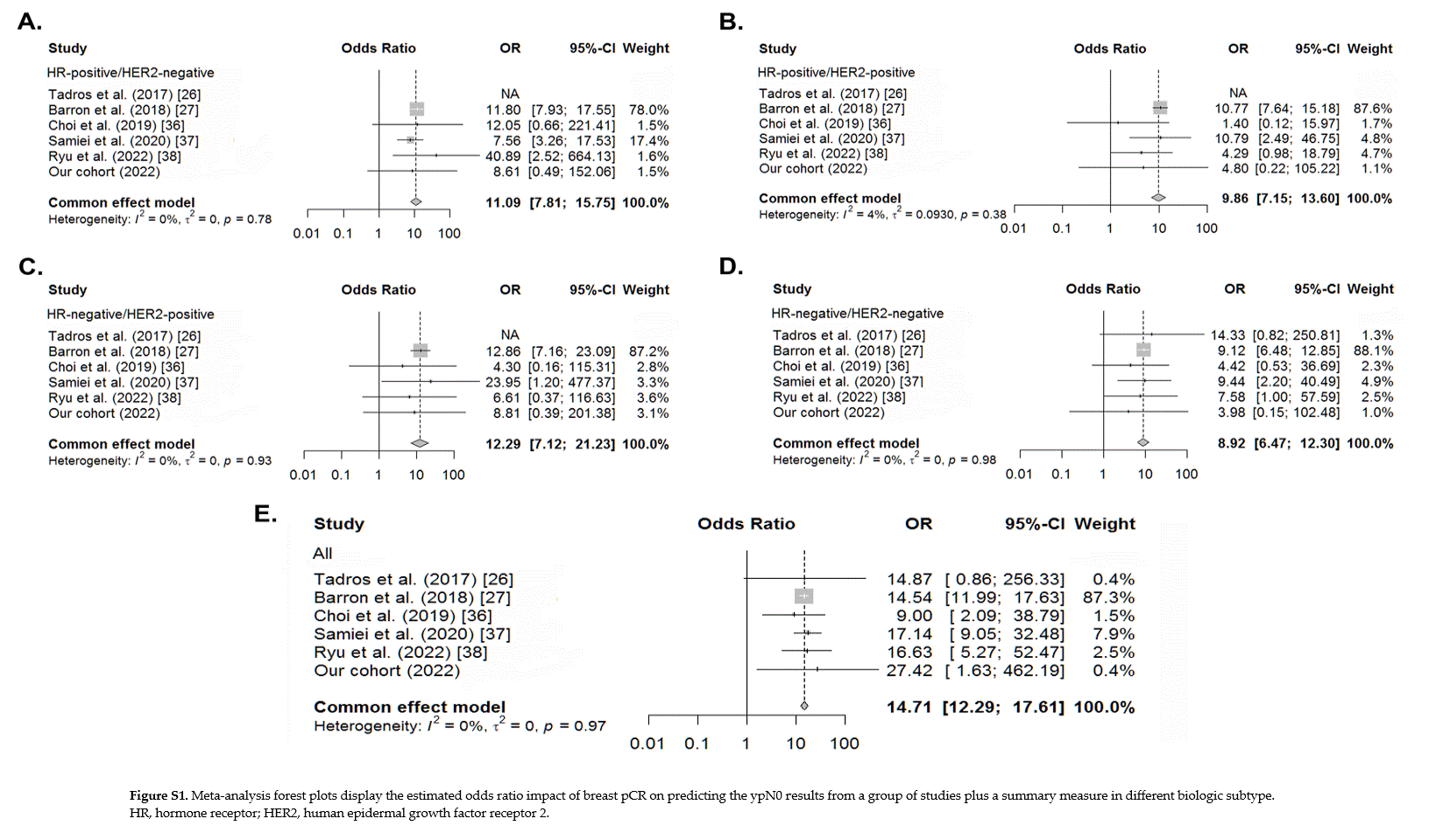

Supplement: Supplementary file 1 [file cancers-14-04451-s001.zip › cancers-1867062-Figure S1.gif]
